# Supplementary material for: Lactiplantibacillus plantarum LPJZ-658 Improves Non-Alcoholic Steatohepatitis by Modulating Bile Acid Metabolism and Gut Microbiota in Mice
Source: Int J Mol Sci. 2023 Sep 12;24(18):13997. doi: 10.3390/ijms241813997 (PMC10531215; doi:10.3390/ijms241813997)
Supplement: Supplementary file 1 [file ijms-24-13997-s001.zip › ijms-2586433-supplementary.pdf]

Supplementary Material for:

Lactiplantibacillus plantarum LPJZ-658 Improves Non-Alcoholic Steatohepatitis by Modulating Bile Acid Metabolism and Gut Microbiota in Mice

Liming Liu [1], Liquan Deng [2], Wei Wei [3], Chunhua Li [1], Yuting Lu [1], Jieying Bai [4], Letian Li [5], Heping Zhang [6], Ningyi Jin [5], Chang Li [5,*] and Cuiqing Zhao [1,*]

MATERIAL AND METHODS

Serum detection

Serum was obtained by centrifuging whole blood samples. Serum levels of alanine aminotransferase (ALT), aspartate aminotransferase (AST), triglyceride (TG), total cholesterol (T-CHO), high-density lipoprotein (HDL), low-density lipoprotein (LDL), non-esterified fatty acids (NEFA), glycerol was measured using ELISA kit (Nanjing Jiancheng Institute of Bioengineering, China). The serum levels of IL-6, TNF-α, and MCP1 were performed using ELISA kits (BD Biosciences, San Diego, CA, USA).

Liver TG and T-CHO assay

Commercial kits were used to measure the TG and TC levels in liver tissues. Briefly, 0.1 g liver tissue (1:9, w/v) was homogenized with 0.9% sodium chloride buffer using a SCIENTZ-48L homogenizer (Ningbo Scientz Biotechnology, Ningbo, China). After centrifugation (2500 rpm, 15 min, 4 °C), the supernatant was subjected to the measurement of TG and T-CHO concentrations (Nanjing Jiancheng Institute of Bioengineering, Nanjing, China).

Histological analysis

The left liver lobe were fixed in 10% buffered formalin, embedded in paraffin, and routinely processed for histological evaluation (5 μm thick). Steatosis and inflammation were assessed by hematoxylin and eosin (H&E) staining (Wuhan Servicebio Technology Co., Ltd., Wuhan, China), and fibrosis was assessed by Sirius Red staining (Wuhan Servicebio Technology Co., Ltd., Wuhan, China). The percentage of fibrosis area in Sirius Red staining was measured by Image J 1.8.0. Two investigators who were blinded to the treatment independently evaluated the slides and assigned scores using the NASH Clinical Research Network histological scoring system [72]. Briefly, the progression of steatohepatitis was assessed using a non-alcoholic fatty liver disease activity score (NAS), which is composed of three scores: steatosis severity (0-3), lobular inflammation (0-2) and hepatocyte ballooning (0-3). In addition, a 5-point scale (0-4) was scored on hepatic fibrosis. Additional frozen sections were sliced into 6 μm thick slices and further confirmed the presence of steatosis using Oil Red O staining.

Quantitative PCR (q-PCR) analysis

The mRNA levels were assessed by q-PCR. In brief, total RNA was isolated using RNAiso Plus (Takara Bio Inc, Kusatsu, Shiga, Japan) and reverse-transcribed into cDNA using a PrimeScript™ RT reagent Kit with gDNA Eraser

(Perfect Real Time) (Takara Bio Inc, Kusatsu, Shiga, Japan) according to the manufacturer's instructions. TB GreenPremix Ex Taq (Tli RNaseH Plus) (Takara Bio Inc, Kusatsu, Shiga, Japan) was used to quantify the PCR amplification products. The expression levels of the target mRNA were measured using ABI StepOnePlus real-time PCR thermocycler (Thermo Fisher Scientific, Waltham, MA, USA). The target mRNA levels were normalized to the levels of the housekeeping gene 18s, which was used as the endogenous control. The sequences of the primers are listed in Table S2.

Western blot analysis

Mouse tissues were lysed with ice-cold lysis buffer for Western blot (Beyotime Biotechnology, Shanghai, China) supplemented with a Protease and a phosphatase inhibitor cocktail (Beyotime Biotechnology, Shanghai, China) and centrifuged at 12,000 rpm for 15 min. The protein concentrations were measured with a BCA kit (TermoFisher Scientific, Rockford, USA). Thirty micrograms of protein were loaded onto SDS-PAGE gels (EpiZyme Biotechnology, Shanghai, China), and the separated proteins transferred onto polyvinylidene difluoride (PVDF) membranes (Millipore, Billerica, MA, USA). The membranes were blocked with 5% skimmed milk, and the blots were incubated overnight with specific antibodies (FAS, SCD1, CPT1, α-SMA, TGF-β, β-actin, Cell Signaling Technologies, Beverly, MA). The secondary antibody was purchased from Cell Signaling Technologies (Cell Signaling Technologies, Beverly, MA). Images were obtained by the Syngene G:box (Gene Company Limited, Hongkong, China) using ECL Western blot detection reagent (Pierce) (TermoFisher Scientific, Rockford, USA).

Metabolite extraction

A 50 µl of serum sample was subjected to precipitation of protein with 150 µl of methanol. The resulting mixture was then centrifuged at 12,000 rpm for 15 min at 4°C, and the supernatant was transferred to a centrifuge tube and lyophilized using a freeze dryer. The lyophilized samples were re-dissolved in 200 ul of methanol/water (1:1), and centrifuged at 15,000rpm for 15min at 4°C. The method for mixing quality control (QC) samples is to mix equal volumes of each sample for method validation.

LC-MS analysis of serum metabolite

Serum sample extracts were analyzed using a U3000 UPLC system coupled to a Q-Orbitrap mass spectrometer (Thermo Fisher Scientific, San Jose, CA, USA) equipped with an electrospray (ESI) ionization source. The samples were performed on a Thermo Scientific Hyperisil Gold chromatographic column (100mm×2.1mm, 3 μm), the column temperature was 40 ℃, and the injection volume was 5 μl. Mobile phase A was 0.1% formic acid in water (Phase A) and 0.1% formic acid in acetonitrile (Phase B) with 0.4ml/min flow rate under the following gradient program: 3%B (0-1min); 3-70%B (1-8min); 70%B (8-10min); 70-90% (10-17min); 90-100% (17-18min); 100% (18-21min); 100-3%B (21-23min); 3%B (23-26min).

Mass spectrometry was operated in both positive and negative ion modes. Profile data was acquired in the 100-1500m/z range. Tandem MS information was obtained under DDMS2 (TOP 3) mode. The acquisition was performed at a resolution of 17,500, with the ramped normalized collision energy of 30, 50, 60. The key parameters of the ion source were set as follows: The capillary voltage is 3.5 Kv in positive ion mode or -3.2 Kv in negative ion mode, sheath gas flow 50 arb, auxiliary gas flow 15 arb, sweep gas flow 2 arb, capillary temperature 350 ℃. Before sample

analysis, the mass spectrometer was calibrated using Pierce™ calibration solution provided by Thermo Scientific (Thermo Fisher Scientific, San Jose CA, USA). All samples were kept at 4 ℃ during analysis.

Data processing and Metabolites identification

The raw LC-MS data was processed using MS-Dial software 4.90 for peak extraction, alignment, and normalization before being exported as a dataset containing the sample code, peak label, and peak intensity. The metabolite identification of MS1 and MS2 data was also performed by searching the data obtained from the database provided by MS-Dial software.

Microbiome analysis

Mice feces were collected and stored at -80 °C. The feces samples were sent to Novogene Co., Ltd. (Beijing, China) for 16S rRNA sequencing under dry ice preservation. The primers 341F and 806R (Table S1) were chosen to amplify the V3-V4 region of the 16S rRNA gene. Novogene Co., Ltd. completed library construction and Illumina sequencing according to the manufacturer's instructions. The data were analyzed using the Novogene Magic Cloud Platform (https://magic.novogene.com).

Table S1 The information of the annotated metabolite.

No.	RT(min)	Detected m/z	Annotation	HMDB ID	Adducts	CON/NASH	Formular
1	0.633	251.15778	Nardosinone	HMDB0255460	[M+H]+	↓*	$C_{15}H_{22}O_3$
2	0.811	262.06717	Oxolinic acid	HMDB0255996	[M+H]+	↓*	$C_{13}H_{11}NO_5$
3	0.816	162.11121	L-Carnitine	HMDB0000062	[M+H]+	↑***	$C_7H_{15}NO_3$
4	0.838	191.03918	4-Nitroquinoline N-oxide	HMDB0246549	[M+H]+	↑**	$C_9H_6N_2O_3$
5	0.839	180.00227	S-Carboxymethyl-L-cysteine	HMDB0029415	[M+H]+	↓***	$C_5H_9NO_4S$
6	0.845	182.11397	Pregabalin	HMDB0014375	[M+Na]+	↑***	$C_8H_{17}NO_2$
7	0.873	397.16837	Mirabegron	HMDB0254748	[M+H]+	↓*	$C_{21}H_{24}N_4O_2S$
8	0.886	309.1268	Estrone	HMDB0000145	[M+K]+	↑*	$C_{18}H_{22}O_2$
9	0.887	310.05417	Cyanidin	HMDB0002708	[M+Na]+	↑*	$C_{15}H_{11}O_6$
10	0.888	505.99362	Adenosine triphosphate	HMDB0000538	[M-H]-	↑**	$C_{10}H_{16}N_5O_{13}P_3$
11	0.889	199.99805	Cysteine-S-sulfate	HMDB0000731	[M-H]-	↑***	$C_3H_7NO_5S_2$
12	0.889	312.05127	Aristolochic acid B	HMDB0248599	[M+H]+	↑***	$C_{16}H_9NO_6$
13	0.895	294.00055	N-Acetylmuramate	HMDB0060493	[M+H]+	↑*	$C_{11}H_{19}NO_8$
14	0.896	162.07497	Aminoadipic acid	HMDB0000510	[M+H]+	↓*	$C_6H_{11}NO_4$
15	0.896	145.04819	Erythritol	HMDB0002994	[M+Na]+	↑*	$C_4H_{10}O_4$
16	0.898	179.05429	D-Mannose	HMDB0000169	[M-H]-	↑**	$C_6H_{12}O_6$
17	0.898	161.04379	Alpha-D-Glucose	HMDB0003345	[M-H-H2O]-	↑**	$C_6H_{12}O_6$
18	0.901	131.03305	Ethylmalonic acid	HMDB0000622	[M-H]-	↑**	$C_5H_8O_4$
19	0.902	233.06157	Butylparaben	HMDB0032575	[M+K]+	↓**	$C_{11}H_{14}O_3$
20	0.906	149.04372	L-Arabinose	HMDB0000646	[M-H]-	↑**	$C_5H_{10}O_5$
21	0.909	151.05934	D-Arabitol	HMDB0000568	[M-H]-	↓***	$C_5H_{12}O_5$
22	0.912	206.0441	Xanthurenic acid	HMDB0000881	[M+H]+	↓*	$C_{10}H_7NO_4$
23	0.921	248.06194	Forchlorfenuron	HMDB0252436	[M+H]+	↓*	$C_{12}H_{10}ClN_3O$

24	0.937	181.03171	Allantoin	HMDB0000462	[M+Na]+	↓**	$C_4H_6N_4O_3$
25	0.944	282.11566	1-Methyladenosine	HMDB0003331	[M+H]+	↓**	$C_{11}H_{15}N_5O_4$
26	0.947	148.05911	L-Glutamic acid	HMDB0000148	[M+H]+	↓***	$C_5H_9NO_4$
27	0.95	143.08017	Piracetam	HMDB0256585	[M+H]+	↓***	$C_6H_{10}N_2O_2$
28	0.951	175.0007	N-Acetylornithine	HMDB0003357	[M+H]+	↑***	$C_7H_{14}N_2O_3$
29	0.952	286.13742	Piperine	HMDB0029377	[M+H]+	↓**	$C_{17}H_{19}NO_3$
30	0.959	120.06488	L-Threonine	HMDB0000167	[M+H]+	↓***	$C_4H_9NO_3$
31	0.961	266.12146	Thiamine	HMDB0000235	[M+K]+	↓***	$C_{12}H_{17}N_4OS$
32	0.962	172.07004	Metronidazole	HMDB0015052	[M+H]+	↓***	$C_6H_9N_3O_3$
33	0.965	436.15503	Fluphenazine	HMDB0014761	[M-H]-	↑***	$C_{22}H_{26}F_3N_3OS$
34	0.968	214.06926	Baclofen	HMDB0014327	[M+H]+	↓***	$C_{10}H_{12}ClNO_2$
35	0.97	277.08765	Clenbuterol	HMDB0015477	[M+H]+	↓***	$C_{12}H_{18}Cl_2N_2O$
36	0.972	158.0799	3-Methylcrotonylglycine	HMDB0000459	[M+H]+	↓**	$C_7H_{11}NO_3$
37	0.973	146.08	4-Acetamidobutanoic acid	HMDB0003681	[M+H]+	↓***	$C_6H_{11}NO_3$
38	0.976	220.07982	O-Succinyl-L-homoserine	HMDB0255868	[M+H]+	↓***	$C_8H_{13}NO_6$
39	0.976	438.16776	Pazopanib	HMDB0256144	[M+H]+	↑***	$C_{21}H_{23}N_7O_2S$
40	0.977	249.10641	Creatinine	HMDB0000562	[2M+Na]+	↓***	$C_4H_7N_3O$
41	0.979	161.07764	3-Methyladipic acid	HMDB0000555	[M+H]+	↑***	$C_7H_{12}O_4$
42	0.979	300.16354	Hydrocodone	HMDB0015091	[M+H]+	↓***	$C_{18}H_{21}NO_3$
43	0.98	177.10097	Serotonin	HMDB0000259	[M+H]+	↑***	$C_{10}H_{12}N_2O$
44	0.98	147.04301	Coumarin	HMDB0001218	[M+H]+	↓***	$C_9H_6O_2$
45	0.98	136.0748	p-Octopamine	HMDB0004825	[M+H-H2O]+	↓***	$C_8H_{11}NO_2$
46	0.981	219.12155	3-Hydroxysebacic acid	HMDB0000350	[M+H]+	↓***	$C_{10}H_{18}O_5$
47	0.981	104.05233	Biuret	HMDB0249282	[M+H]+	↓***	$C_2H_5N_3O_2$
48	0.982	273.11688	Naringenin	HMDB0002670	[M+H]+	↓***	$C_{15}H_{12}O_5$

49	0.983	115.04951	Dihydrouracil	HMDB0000076	[M+H]+	↓***	$C_4H_6N_2O_2$
50	0.984	158.08046	Isovalerylglycine	HMDB0000678	[M-H]-	↑***	$C_7H_{13}NO_3$
51	0.986	179.04724	Cysteinylglycine	HMDB0000078	[M+H]+	↓**	$C_5H_{10}N_2O_3S$
52	0.986	137.04459	Hypoxanthine	HMDB0000157	[M+H]+	↑**	$C_5H_4N_4O$
53	0.99	309.09213	Clobenpropit	HMDB0250329	[M+H]+	↓**	$C_{14}H_{17}ClN_4S$
54	0.995	154.04291	4-Hydroxyproline	HMDB0000725	[M+Na]+	↑***	$C_5H_9NO_3$
55	1.002	305.04587	Calycin	HMDB0249565	[M-H]-	↑***	$C_{18}H_{10}O_5$
56	1.012	338.02753	Tenoxicam	HMDB0014612	[M+H]+	↑*	$C_{13}H_{11}N_3O_4S_2$
57	1.013	308.04501	Clopidogrel carboxylic acid	HMDB0250357	[M+H]+	↑***	$C_{15}H_{14}ClNO_2S$
58	1.041	140.06685	5-Aminopentanoic acid	HMDB0003355	[M+Na]+	↑***	$C_5H_{11}NO_2$
59	1.048	263.04315	2',2'-Difluorodeoxyuridine	HMDB0060727	[M-H]-	↓***	$C_9H_{10}F_2N_2O_5$
60	1.141	198.99986	Dodecanoic acid	HMDB0000638	[M-H]-	↓**	$C_{12}H_{24}O_2$
61	1.152	245.04166	2,2',4,4'-Tetrahydroxybenzophenone	HMDB0244537	[M-H]-	↓**	$C_{13}H_{10}O_5$
62	1.155	258.10815	Glycerophosphocholine	HMDB0000086	[M+H]+	↓**	$C_8H_{21}NO_6P$
63	1.161	304.99841	Chloramphenicol	HMDB0014589	[M+H-H2O]+	↓**	$C_{11}H_{12}Cl_2N_2O_5$
64	1.163	127.03809	Maltol	HMDB0030776	[M+H]+	↓**	$C_6H_6O_3$
65	1.365	259.02145	Fructose 6-phosphate	HMDB0000124	[M-H]-	↓**	$C_6H_{13}O_9P$
66	1.372	186.0858	Secnidazole	HMDB0258202	[M+H]+	↓***	$C_7H_{11}N_3O_3$
67	1.411	218.13742	Propionylcarnitine	HMDB0000824	[M+H]+	↑*	$C_{10}H_{19}NO_4$
68	1.414	166.0564	Methionine sulfoxide	HMDB0002005	[M+H]+	↓***	$C_5H_{11}NO_3S$
69	1.417	142.0237	O-Phosphoethanolamine	HMDB0000224	[M+H]+	↓***	$C_2H_8NO_4P$
70	1.421	182.07993	L-Tyrosine	HMDB0000158	[M+H]+	↓***	$C_9H_{11}NO_3$
71	1.455	215.0312	Methoxsalen	HMDB0014693	[M-H]-	↑***	$C_{12}H_8O_4$
72	1.466	229.0107	D-Ribose 5-phosphate	HMDB0001548	[M-H]-	↑***	$C_5H_{11}O_8P$
73	1.504	268.10172	Adenosine	HMDB0000050	[M+H]+	↓***	$C_{10}H_{13}N_5O_4$

74	1.72	130.08546	L-Isoleucine	HMDB0000172	[M-H]-	↑***	$C_6H_{13}NO_2$
75	1.871	165.05347	4-Hydroxycinnamic acid	HMDB0002035	[M+H]+	↓*	$C_9H_8O_3$
76	1.872	147.07512	L-Glutamine	HMDB0000641	[M+H]+	↓***	$C_5H_{10}N_2O_3$
77	2.047	113.03402	Uracil	HMDB0000300	[M+H]+	↓***	$C_4H_4N_2O_2$
78	2.121	152.05551	Guanine	HMDB0000132	[M+H]+	↑***	$C_5H_5N_5O$
79	2.253	119.03443	Methylmalonic acid	HMDB0000202	[M+H]+	↑***	$C_4H_6O_4$
80	2.281	267.07242	Inosine	HMDB0000195	[M-H]-	↑***	$C_{10}H_{12}N_4O_5$
81	2.944	229.11688	Prolylhydroxyproline	HMDB0006695	[M+H]+	↓***	$C_{10}H_{16}N_2O_4$
82	3.019	291.15353	Androsterone	HMDB0000031	[M+H]+	↓**	$C_{19}H_{30}O_2$
83	3.039	153.03961	Xanthine	HMDB0000292	[M+H]+	↓**	$C_5H_4N_4O_2$
84	3.129	279.09857	gamma-Glutamylmethionine	HMDB0034367	[M+H]+	↑**	$C_{10}H_{18}N_2O_5S$
85	3.17	205.08051	Levamisole	HMDB0014986	[M+H]+	↓**	$C_{11}H_{12}N_2S$
86	3.338	311.12076	gamma-Glutamyltyrosine	HMDB0011741	[M+H]+	↓**	$C_{14}H_{18}N_2O_6$
87	3.368	207.08597	Diphenylcyclopropenone	HMDB0251457	[M+H]+	↑*	$C_{15}H_{10}O$
88	3.595	227.07761	L-Tryptophan	HMDB0000929	[M+Na]+	↓*	$C_{11}H_{12}N_2O_2$
89	3.595	160.07455	Indoleacetaldehyde	HMDB0001190	[M+H]+	↓*	$C_{10}H_9NO$
90	3.595	203.08003	vasicinone	HMDB0259770	[M+H]+	↓*	$C_{11}H_{10}N_2O_2$
91	3.692	117.05398	3-Hydroxyisovaleric acid	HMDB0000754	[M-H]-	↓**	$C_5H_{10}O_3$
92	3.752	349.14868	Ajugol	HMDB0248074	[M+H]+	↓**	$C_{15}H_{24}O_9$
93	4.521	372.16409	Trazodone	HMDB0014794	[M+H]+	↓**	$C_{19}H_{22}ClN_5O$
94	4.543	226.14162	Terbutaline	HMDB0015009	[M+H]+	↑**	$C_{12}H_{19}NO_3$
95	4.595	319.13898	Mycophenolic acid	HMDB0015159	[M-H]-	↑**	$C_{17}H_{20}O_6$
96	4.673	178.0495	Hippuric acid	HMDB0000714	[M-H]-	↑***	$C_9H_9NO_3$
97	4.673	246.0369	Emtricitabine	HMDB0015017	[M-H]-	↑***	$C_8H_{10}FN_3O_3S$
98	4.872	134.02606	Thioproline	HMDB0062164	[M+H]+	↑**	$C_4H_7NO_2S$

99	5.778	137.13132	Myrcene	HMDB0038169	[M+H]+	↓***	$C_{10}H_{16}$
100	5.779	179.14194	menthol	HMDB0003352	[M+Na]+	↓***	$C_{10}H_{20}O$
101	5.823	123.07955	1-Phenylethanol	HMDB0032619	[M+H]+	↓***	$C_8H_{10}O$
102	5.867	118.06453	Norvaline	HMDB0013716	[M+H]+	↓***	$C_5H_{11}NO_2$
103	5.868	146.05907	1H-Indole-3-carboxaldehyde	HMDB0029737	[M+H]+	↓**	C_9H_7NO
104	6.035	130.06435	3-Methylindole	HMDB0000466	[M-H]-	↑***	C_9H_9N
105	6.571	318.17123	Cocaethylene	HMDB0041860	[M+H]+	↓***	$C_{18}H_{23}NO_4$
106	6.588	407.27899	Cholic acid	HMDB0000619	[M-H]-	↓***	$C_{24}H_{40}O_5$
107	6.601	204.06403	2,8-Quinolinediol	HMDB0240311	[M+K]+	↑***	$C_{11}H_9NO_3$
108	6.604	176.06944	Guanidinosuccinic acid	HMDB0003157	[M+H]+	↑***	$C_5H_9N_3O_4$
109	6.659	168.02863	2-Furoylglycine	HMDB0000439	[M-H]-	↑**	$C_7H_7NO_4$
110	6.939	130.99068	L-Asparagine	HMDB0000168	[M-H]-	↓***	$C_4H_8N_2O_3$
111	6.952	464.29968	Glycocholic acid	HMDB0000138	[M-H]-	↓**	$C_{26}H_{43}NO_6$
112	7.094	284.2919	Guanosine	HMDB0000133	[M+H]+	↓***	$C_{10}H_{13}N_5O_5$
113	7.276	207.13748	3-(3,4-Dimethoxyphenyl)-2-propenoic acid	HMDB0034315	[M-H]-	↓**	$C_{11}H_{12}O_4$
114	7.282	373.27158	Hyocholic acid	HMDB0000760	[M+H-2H2O]+	↓**	$C_{24}H_{40}O_5$
115	7.467	583.25146	Biliverdin	HMDB0001008	[M+H]+	↓*	$C_{33}H_{34}N_4O_6$
116	7.574	424.33948	Linoleyl carnitine	HMDB0006469	[M+H]+	↓**	$C_{25}H_{45}NO_4$
117	7.632	351.2207	Griseofulvin	HMDB0014544	[M-H]-	↓***	$C_{17}H_{17}ClO_6$
118	7.723	209.13089	Pilocarpine	HMDB0015217	[M+H]+	↓***	$C_{11}H_{16}N_2O_2$
119	7.804	391.28473	Deoxycholic acid	HMDB0000626	[M-H]-	↓**	$C_{24}H_{40}O_4$
120	7.896	516.29572	Candoxatril	HMDB0014754	[M+H]+	↓***	$C_{29}H_{41}NO_7$
121	7.917	462.26361	Taurocholic acid	HMDB0000036	[M+H-3H2O]+	↓***	$C_{26}H_{45}NO_7S$
122	8.02	243.15891	1,11-Undecanedicarboxylic acid	HMDB0002327	[M-H]-	↓***	$C_{13}H_{24}O_4$
123	8.078	303.22885	Linoleic acid	HMDB0000673	[M+Na]+	↓***	$C_{18}H_{32}O_2$

124	8.196	325.25089	Uridine 5'-monophosphate	HMDB0000288	[M+H]+	↓*	$C_9H_{13}N_2O_9P$
125	8.364	405.26352	3-Oxocholic acid	HMDB0000502	[M-H]-	↓***	$C_{24}H_{38}O_5$
126	8.7	227.20045	Myristic acid	HMDB0000806	[M-H]-	↓***	$C_{14}H_{28}O_2$
127	8.836	382.26816	Sphinganine 1-phosphate	HMDB0001383	[M+H]+	↓***	$C_{18}H_{40}NO_5P$
128	8.876	285.12466	HMDB0249370	HMDB0249370	[M-H]-	↑***	$C_{16}H_{14}O_5$
129	8.973	495.18594	Harpagoside	HMDB0253053	[M+H]+	↓**	$C_{24}H_{30}O_{11}$
130	9.013	391.28455	Chenodeoxycholic acid	HMDB0000518	[M-H]-	↓**	$C_{24}H_{40}O_4$
131	9.023	184.07219	Phosphorylcholine	HMDB0001565	[M]+	↓***	$C_5H_{15}NO_4P$
132	9.058	285.20572	Kaempferol	HMDB0005801	[M-H]-	↓***	$C_{15}H_{10}O_6$
133	9.063	518.32123	LysoPC(18:3(6Z,9Z,12Z)/0:0)	HMDB0010387	[M+H]+	↓***	$C_{26}H_{48}NO_7P$
134	9.209	279.19583	12S-HHT	HMDB0012535	[M-H]-	↓**	$C_{17}H_{28}O_3$
135	9.421	372.18103	Demecolcine	HMDB0250401	[M+H]+	↓***	$C_{21}H_{25}NO_5$
136	9.533	496.33539	LysoPC(16:0)	HMDB0010382	[M+H]+	↓***	$C_{24}H_{50}NO_7P$
137	9.65	452.27701	LysoPE(0:0/16:0)	HMDB0011473	[M-H]-	↓**	$C_{21}H_{44}NO_7P$
138	9.955	454.28955	LysoPE(16:0/0:0)	HMDB0011503	[M+H]+	↓***	$C_{21}H_{44}NO_7P$
139	10.095	480.30505	LysoPE(0:0/18:1(11Z))	HMDB0011475	[M+H]+	↓***	$C_{23}H_{46}NO_7P$
140	10.201	145.10335	5-Hydroxylysine	HMDB0000450	[M+H-H2O]+	↓*	$C_6H_{14}N_2O_3$
141	10.36	219.13609	Isoleucyl-Serine	HMDB0028916	[M+H]+	↓***	$C_9H_{18}N_2O_4$
142	10.583	478.29321	LysoPE(0:0/18:1(9Z))	HMDB0011476	[M-H]-	↓***	$C_{23}H_{46}NO_7P$
143	10.591	481.30777	Emetine	HMDB0251773	[M+H]+	↓***	$C_{29}H_{40}N_2O_4$
144	10.629	522.35077	LysoPC(18:1(9Z))	HMDB0002815	[M+H]+	↓***	$C_{26}H_{52}NO_7P$
145	10.657	261.14417	Isoleucyl-Glutamate	HMDB0028906	[M+H]+	↑**	$C_{11}H_{20}N_2O_5$
146	11.021	538.27802	taurohyocholic acid	HMDB0011637	[M+Na]+	↓***	$C_{26}H_{45}NO_7S$
147	11.876	376.15503	Haloperidol	HMDB0014645	[M+H]+	↓**	$C_{21}H_{23}ClFNO_2$
148	11.972	389.22992	Digoxigenin	HMDB0060731	[M-H]-	↓***	$C_{23}H_{34}O_5$

149	11.978	303.23099	Arachidonic acid	HMDB0001043	[M-H]-	↓***	$C_{20}H_{32}O_2$
150	12.089	369.31229	Methoxyfenozide	HMDB0254547	[M+H]+	↓*	$C_{22}H_{28}N_2O_3$
151	12.191	315.22974	Progesterone	HMDB0001830	[M+H]+	↓***	$C_{21}H_{30}O_2$
152	12.313	782.56628	PC(16:0/18:1(9Z))	HMDB0007972	[M+Na]+	↑**	$C_{42}H_{82}NO_8P$
153	12.486	552.39862	Aliskiren	HMDB0015387	[M+H]+	↓***	$C_{30}H_{53}N_3O_6$
154	12.737	159.11552	D-Limonene	HMDB0003375	[M+Na]+	↓***	$C_{10}H_{16}$
155	12.737	297.18216	Exemestane	HMDB0015125	[M+H]+	↓***	$C_{20}H_{24}O_2$
156	12.738	265.15613	Phenylalanyl-Valine	HMDB0029008	[M+H]+	↓***	$C_{14}H_{20}N_2O_3$
157	12.742	199.1424	Guaiazulene	HMDB0036648	[M+H]+	↓***	$C_{15}H_{18}$
158	13.213	279.23001	alpha-Linolenic acid	HMDB0001388	[M+H]+	↑***	$C_{18}H_{30}O_2$
159	13.263	355.28058	MG(18:2(9Z,12Z)/0:0/0:0)	HMDB0011568	[M+H]+	↑*	$C_{21}H_{38}O_4$
160	13.709	391.28464	Hyodeoxycholic acid	HMDB0000733	[M-H]-	↓***	$C_{24}H_{40}O_4$
161	14.318	339.25052	Kirenol	HMDB0253803	[M+H]+	↓**	$C_{20}H_{34}O_4$
162	14.388	385.34271	Cholestenone	HMDB0000921	[M+H]+	↓**	$C_{27}H_{44}O$
163	14.469	225.14799	Methyl dihydrojasmonate	HMDB0031740	[M-H]-	↓***	$C_{13}H_{22}O_3$
164	14.895	307.26108	Dihomo-gamma-linolenic acid	HMDB0002925	[M+H]+	↓***	$C_{20}H_{34}O_2$
165	14.97	328.31827	Stearoylethanolamide	HMDB0013078	[M+H]+	↓**	$C_{20}H_{41}NO_2$
166	15.526	731.60016	SM(d18:1/18:0)	HMDB0001348	[M+H]+	↑*	$C_{41}H_{83}N_2O_6P$
167	16.095	315.25235	Octadecanedioic acid	HMDB0000782	[M+H]+	↓***	$C_{18}H_{34}O_4$
168	16.142	293.24478	Androstanediol	HMDB0000495	[M+H]+	↓**	$C_{19}H_{32}O_2$
169	17.107	161.09462	D-Alanyl-D-alanine	HMDB0003459	[M+H]+	↑**	$C_6H_{12}N_2O_3$
170	18.035	249.15575	Pindolol	HMDB0015095	[M+H]+	↑*	$C_{14}H_{20}N_2O_2$
171	18.103	301.11624	Phenmedipham	HMDB0256405	[M+H]+	↓**	$C_{16}H_{16}N_2O_4$
172	18.142	319.12607	Tebupirimfos	HMDB0258766	[M+H]+	↓**	$C_{13}H_{23}N_2O_3PS$
173	18.146	362.13998	Papaverine	HMDB0015245	[M+H]+	↓**	$C_{20}H_{21}NO_4$

174	19.1	311.29474	Arachidic acid	HMDB0002212	[M-H]-	↓***	$C_{20}H_{40}O_2$
175	20.233	112.98373	Acetylenedicarboxylate	HMDB0247933	[M-H]-	↑*	$C_4H_2O_4$
176	22.378	628.35498	Bulleyaconitine A	HMDB0249438	[M+H]+	↑***	$C_{35}H_{49}NO_9$
177	22.396	168.98746	Dihydroxyacetone phosphate	HMDB0001473	[M-H]-	↑***	$C_3H_7O_6P$
178	23.948	126.99928	Cyclohexanecarboxylic acid	HMDB0031342	[M-H]-	↓***	$C_7H_{12}O_2$

*p < 0.05, **p < 0.01, ***p < 0.001 NASH vs CON. "↑" means up-regulate. "↓" means down-regulate.

Table S2: Primer Sequences for q-PCR analysis

Gene	Source	Sequences (Forward/Reverse 5'-3')	
α-SMA	Mouse	CTGACAGAGGCACCACTGAA	GAAGGAATAGCCACGCTCAG
CD36	Mouse	GAACCACTGCTTTCAAAAACTGG	TGCTGTTCTTTGCCACGTCA
ChREBP	Mouse	CTGGGGACCTAAACAGGAGC	GAAGCCACCCTATAGCTCCC
Col1a1	Mouse	GTGTGGAGCAACATGTGGAACTCTA	TTGGTTCAGCCACTGCCGTA
CPT1	Mouse	CCAGGCTACAGTGGGACA	GAACTTGCCCATGTCCTTGT
FAS	Mouse	TGGGTTCTAGCCAGCAGAGT	ACCACCAGAGACCGTTATGC
IL-1β	Mouse	TTCATCTTTGAAGAAGAGCCCAT	TCGGAGCCTGTAGTGCAGTT
IL-6	Mouse	TGGAAATGAGAAAAGAGTTGTGC	CCAGTTTGGTAGCATCCATCA
PGC-1α	Mouse	AGACAAATGTGCTTCCAAAAAGAA	GAAGAGATAAAGTTGGTTTGGC
PPARα	Mouse	AGAGCCCCATCTGTCCTCTC	ACTGGTAGTCTGCAAAACCAAA
TNFα	Mouse	CACCACCATCAAGGACTCAA	AGGCAACCTGACCACTCTCC
SREBP1c	Mouse	GCGGAGCCATGGATTGCA	CTCTTCCTTGATACCAGGCCC
TGF-β	Mouse	GTGTGGAGCAACATGTGGAACTCTA	TTGGTTCAGCCACTGCCGTA
18s	Mouse	CTAACCCGTTGAACCCCATT	CCATCCAATCGGTAGTAGCG
341F		CCTAYGGGRBGCASCAG	
806R		GGACTACNNGGGTATCTAAT	

Figure S1

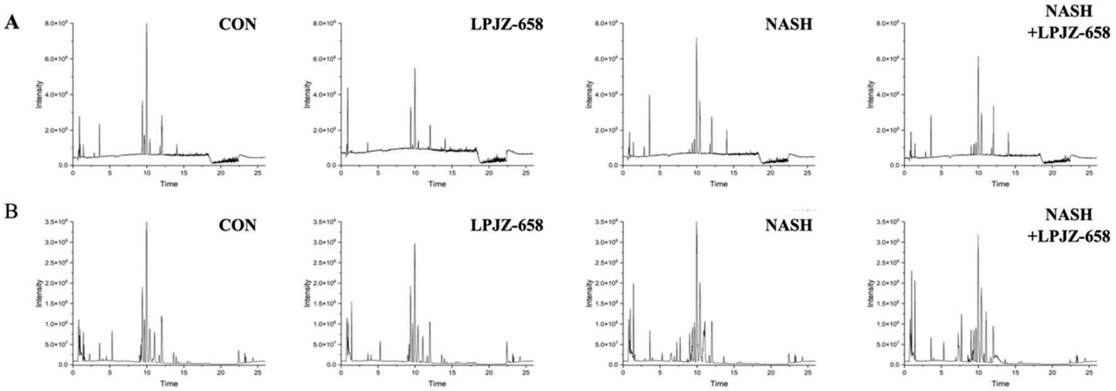

A
CON
LPJZ-658
NASH
NASH +LPJZ-658
B
CON
LPJZ-658
NASH
NASH +LPJZ-658
Intensity
Time

Figure S2

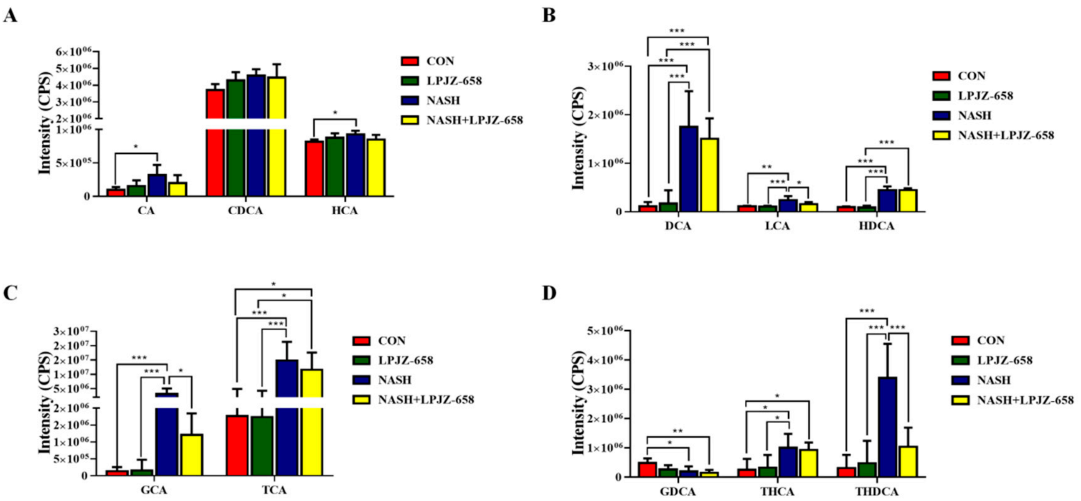
A
Intensity (CPS)
CA
CDCA
HCA
CON
LPJZ-658
NASH
NASH+LPJZ-658
B
Intensity (CPS)
DCA
LCA
HDCA
CON
LPJZ-658
NASH
NASH+LPJZ-658
C
Intensity (CPS)
GCA
TCA
CON
LPJZ-658
NASH
NASH+LPJZ-658
D
Intensity (CPS)
GDCA
THCA
THDCA
CON
LPJZ-658
NASH
NASH+LPJZ-658

Figure S3

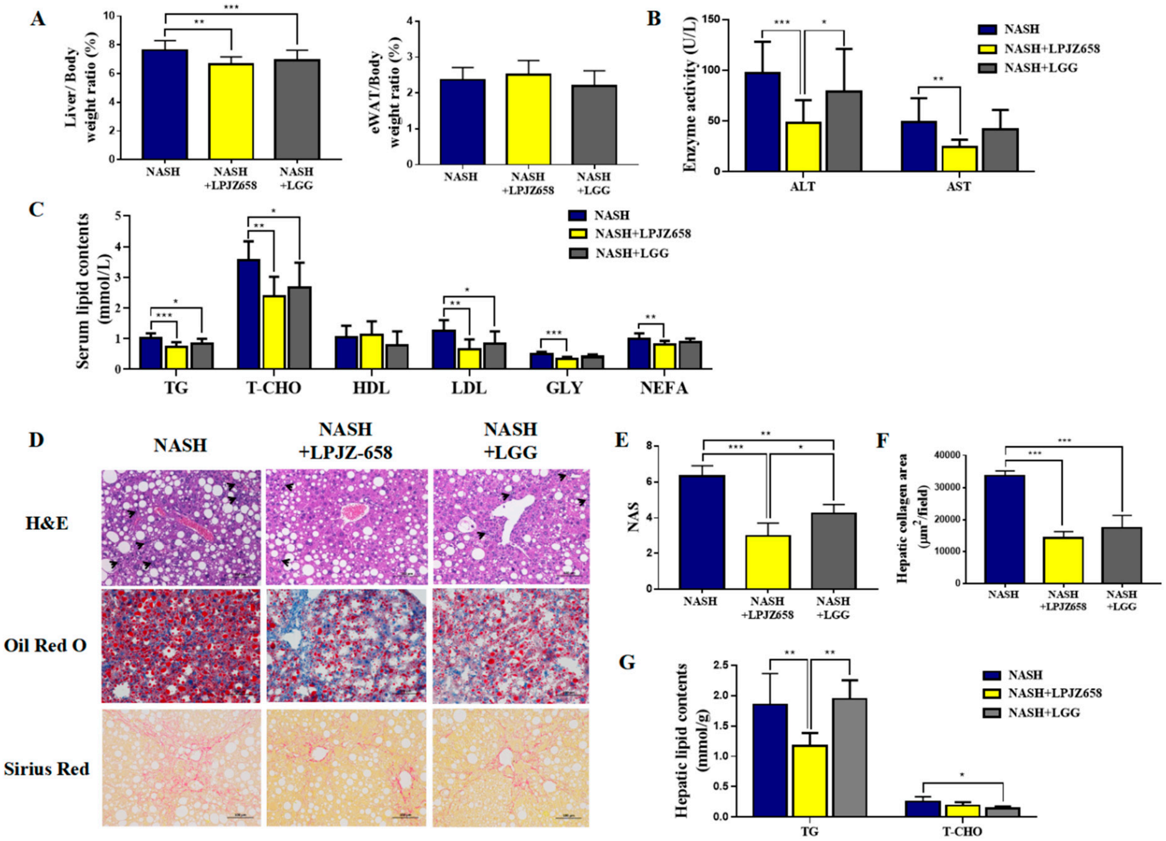
A
Liver/ Body weight ratio (%)
eWAT/Body weight ratio (%)
NASH
NASH +LPJZ658
NASH +LGG
B
Enzyme activity (U/L)
ALT
AST
NASH
NASH+LPJZ658
NASH+LGG
C
Serum lipid contents (mmol/L)
TG
T-CHO
HDL
LDL
GLY
NEFA
D
NASH
NASH +LPJZ-658
NASH +LGG
H&E
Oil Red O
Sirius Red
E
NAS
F
Hepatic collagen area (μm²/field)
G
Hepatic lipid contents (mmol/g)

SUPPLEMENTARY FIGURE LEGENDS

Figure S1. Representative base peak chromatograms of serum samples collected from 50% methanol in water extracts (A, ESI+ and B, ESI-).

Figure S2. Quantitative intensity determination (A) primary bile acids (CA, CDCA and HCA), (B) secondary bile acids (DCA, LAC, and HDCA), (C) conjugated primary bile acids (GCA and TCA) and (D) conjugated secondary bile acids (GDCA, THCA, and THDCA) in serum samples. Data presented indicate the mean ± SEM (* $p < 0.05$, ** $p < 0.01$, and *** $p < 0.001$).

Figure S3. Tissue weight ratio and metabolic parameters in experimental groups subjected to PBS, LPJZ-658, and LGG. Mice were fed a WD/CCl4 diet for 8 weeks and then treated with 1×10^9 CFU/day/mouse LPJZ-658 or Lactobacillus rhamnosus GG (LGG) by oral gavage with continuous WD/CCl4 diet feeding for another 4 weeks. (A) Liver-to-body weight ratio (left), eWAT-to-body weight ratio (right) of mouse. (B) Serum levels of ALT and AST. (C) Serum TG, T-CHO, HDL, LDL, GLY, and NEFA. (D) Representative images of H&E, Oil Red O and Sirius Red staining of liver sections from the indicated mice. Scale bar, 100 μm. (E) NAFLD activity score (NAS). (F) Quantitation of Sirius Red staining. (G) Lipid (TG and T-CHO) levels in the hepatic. Data presented indicate the mean ± SEM (* $p < 0.05$, ** $p < 0.01$, and *** $p < 0.001$).
